# Salvage of Side Branch by Provisional “TAP Technique” Using Absorb™ Bioresorbable Vascular Scaffolds for Bifurcation Lesions: First Case Reports with Technical Considerations

**DOI:** 10.1002/ccd.25444

**Published:** 2014-02-15

**Authors:** Ashok Seth, G Sengottuvelu, V Ravisekar

**Affiliations:** 1 Department of Interventional Cardiology, Fortis Escorts Heart InstituteNew Delhi, India; 2 Department of Interventional Cardiology, Apollo HospitalsChennai, Tamil Nadu, India

**Keywords:** coronary artery disease, optical coherence tomography, percutaneous coronary intervention, stent, bioabsorbable, stenting technique

## Abstract

Recent technological developments have led to the development of Absorb™ bioresorbable vascular scaffold (BVS) [Abbott Vascular, Santa Clara, USA] for percutaneous treatment of coronary artery disease by percutaneous coronary intervention (PCI). The BVS is now approved for use in many countries but experience in bifurcation lesions is limited and largely unreported and concerns still exist about its use across major side branches. We report for the first time, the successful use of the “T and Protrusion” (TAP) technique of deploying BVS into the side branch (SB) through the struts of main branch (MB) BVS to salvage a suboptimal result and threatened closure of a SB in three cases when treating bifurcation lesions with a planned single BVS strategy. The TAP technique was successful in all cases and there were no complications. All patients continue to do well at short-term follow-up. This case report provides information regarding the feasibility as well as technical and procedural insights when using BVS for bifurcation lesions. © 2014 Wiley Periodicals, Inc.

## INTRODUCTION

The concept of a fully absorbable drug eluting stent (DES), which may have potential advantages over a permanent metallic implant, has led to the development of the first commercially available everolimus eluting fully bioresorbable vascular scaffold (BVS) made of poly lactic acid called Absorb™ (BVS) [Abbott Vascular, Santa Clara, USA] [1]. After the first human trials [2], the BVS has been approved for clinical use in many countries worldwide. However, its experience in “real world” cases is limited. Because it is made of polymer and not metal, it has restricted expansion characteristics and has a larger profile (157 μm strut thickness). This has led to concerns regarding its trackability, deliverability and performance in complex lesions. It is recommended to avoid using BVS across a side branch (SB) ≥ 2 mm in diameter (Instructions for Use Manual 2012, Abbott Vascular, Santa Clara). This is based on concerns and uncertainties related to large profile struts covering the SB orifice, difficulty in accessing the SB through the thick struts of the MB scaffold, the possibility of polymer strut fracture while accessing and dilating SB through the MB BVS, and the possibility of MB BVS disruption if the SB had a suboptimal result requiring a bailout two stent strategy. Mechanical disruption of device or suboptimal result could also lead to serious adverse outcomes of stent thrombosis and restenosis.

We report the use of T and Protrusion (TAP) technique in three cases while treating bifurcation lesions with provisional single BVS stent strategy. The TAP technique was successful in all cases and there were no complications. Two of the three patients had an optical coherence tomography (OCT) imaging at the end of procedure, which showed optimal result at the bifurcation and no disruption of either the MB or SB scaffold. All patients continue to do well at short-term follow-up.

## CASE REPORTS

### Case I

**Fig. 1:**
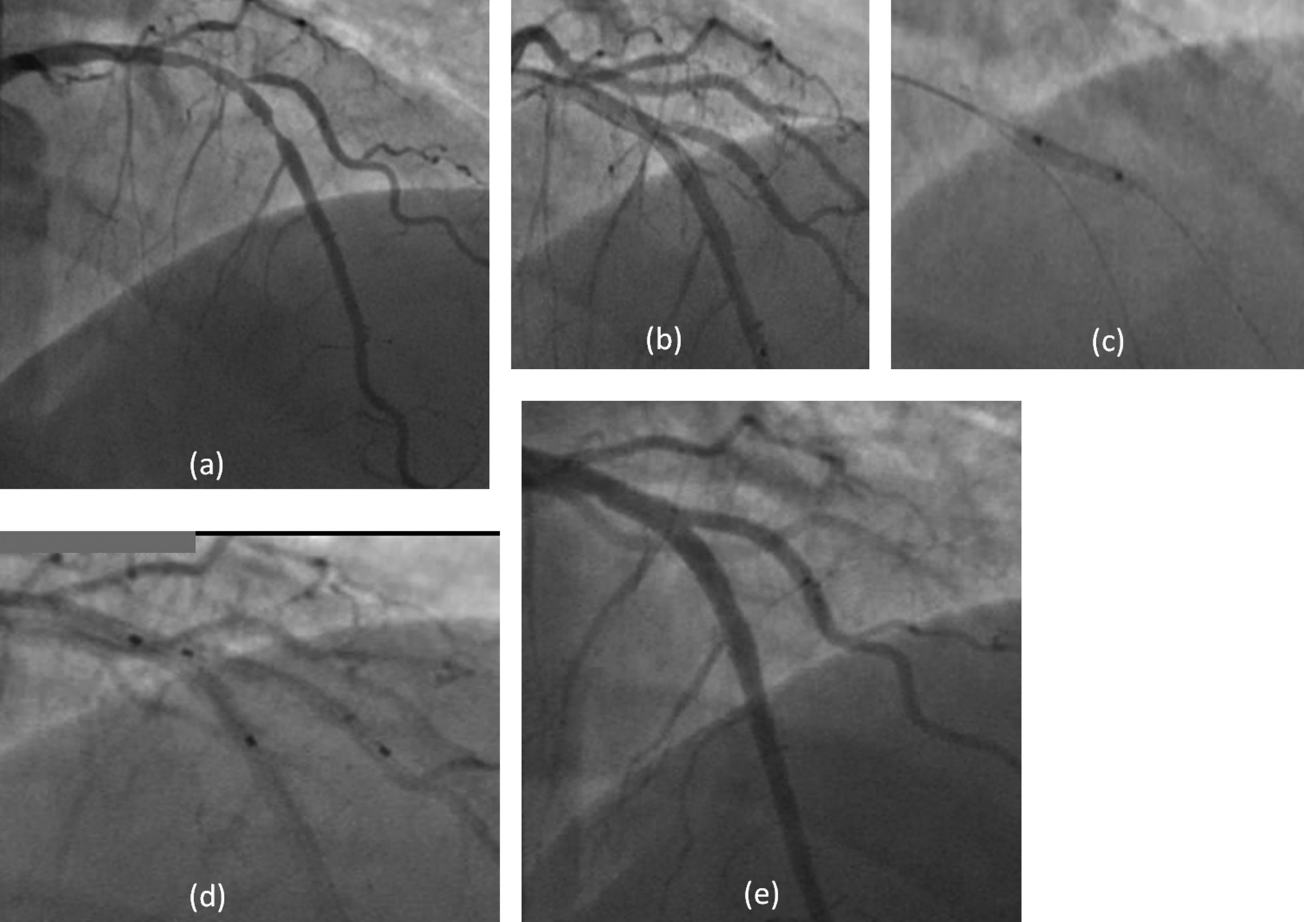
Case 1. (a) RAO cranial projection showing LAD/D1 bifurcation disease; (b) following implantation of BVS in LAD, the ostium of D1 is severely stenosed and dissected; (c) dilatation of D1 through struts of MB BVS; (d) placement of BVS in D1 through struts of LAD BVS; (e) final result.

**Fig. 2:**
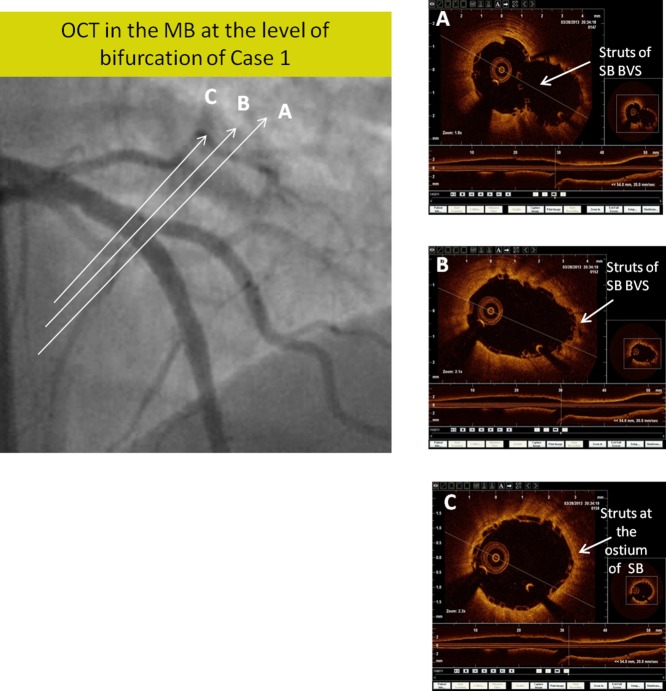
Case 1. A: At the bifurcation carina/distal edge of bifurcation demonstrating struts of SB BVS protruding into the MB a small neocarina, consistent with “TAP”; B: at the centre of bifurcation, no struts across the SB with good cirumferential apposition of struts in the MB as well as SB; C: at the proximal edge of the bifurcation showing good coverage of SB ostium as well as MB, no protrusion, no geographical miss, and no MB device disruption.

A 62-year-old diabetic and hypertensive female suffered a non ST elevation anterior myocardial infarction 4 days prior to admission. Angiography demonstrated bifurcation lesion visually estimated 80% stenosis of the left anterior descending (LAD) artery and 1st diagonal (D1) branch (Medina classification 0, 0, 1). The LAD was 3 mm in diameter and D1 was 2.5 mm in diameter (Fig. 1a). Both LAD and D1 were wired with floppy wires (Balance Middle weight Universal (BMW), Abbott Vascular, Santa Clara, USA). The LAD was predilated with 2.75 mm × 15 mm non-compliant (NC) balloon at 18 atm. D1 ostium was predilated with 2.0 mm × 12 mm NC balloon at 12 atm. LAD was then stented with 3.0 mm × 28 mm BVS at 10 atm and post dilated with 3.0 mm × 15 mm NC balloon at 22 atm. Following this, the D1 ostium was noted to be 90% stenosed (narrowed) with dissection (Fig. 1b). The struts of the MB BVS were crossed with a BMW wire (Abbott Vascular, Santa Clara, USA) into the D1. The D1 ostium was crossed through the struts of MB BVS and dilated sequentially using a 2.0 mm × 10 mm and 2.5 mm × 12 mm balloon up to 14 atm (Fig. 1c). The D1 ostium still revealed a significant residual stenosis with recoil and dissection. To salvage this, a 2.5 mm × 18 mm BVS was passed over the D1 wire through the struts of MB BVS into the D1. The proximal balloon marker of the SB BVS was placed just proximal to the SB ostium, such that the proximal scaffold marker was at the ostium and it was deployed at 10 atm (Fig. 1d). Further post-dilation of SB BVS was performed using 2.5 mm × 12 mm NC balloon to 20 atm followed by dilatation of MB BVS with a 3.0 mm × 12 mm NC balloon to 20 atm. Simultaneous snuggle balloon dilatation (as described in the discussion) was performed for final optimization of result. OCT imaging was performed in the MB as well as SB and showed good coverage and optimization of scaffold geometry at the bifurcation with no disruptions (Fig. 2). The patient was discharged uneventfully after 2 days and continues to do well at 10-months of clinical follow-up.

**Fig. 3:**
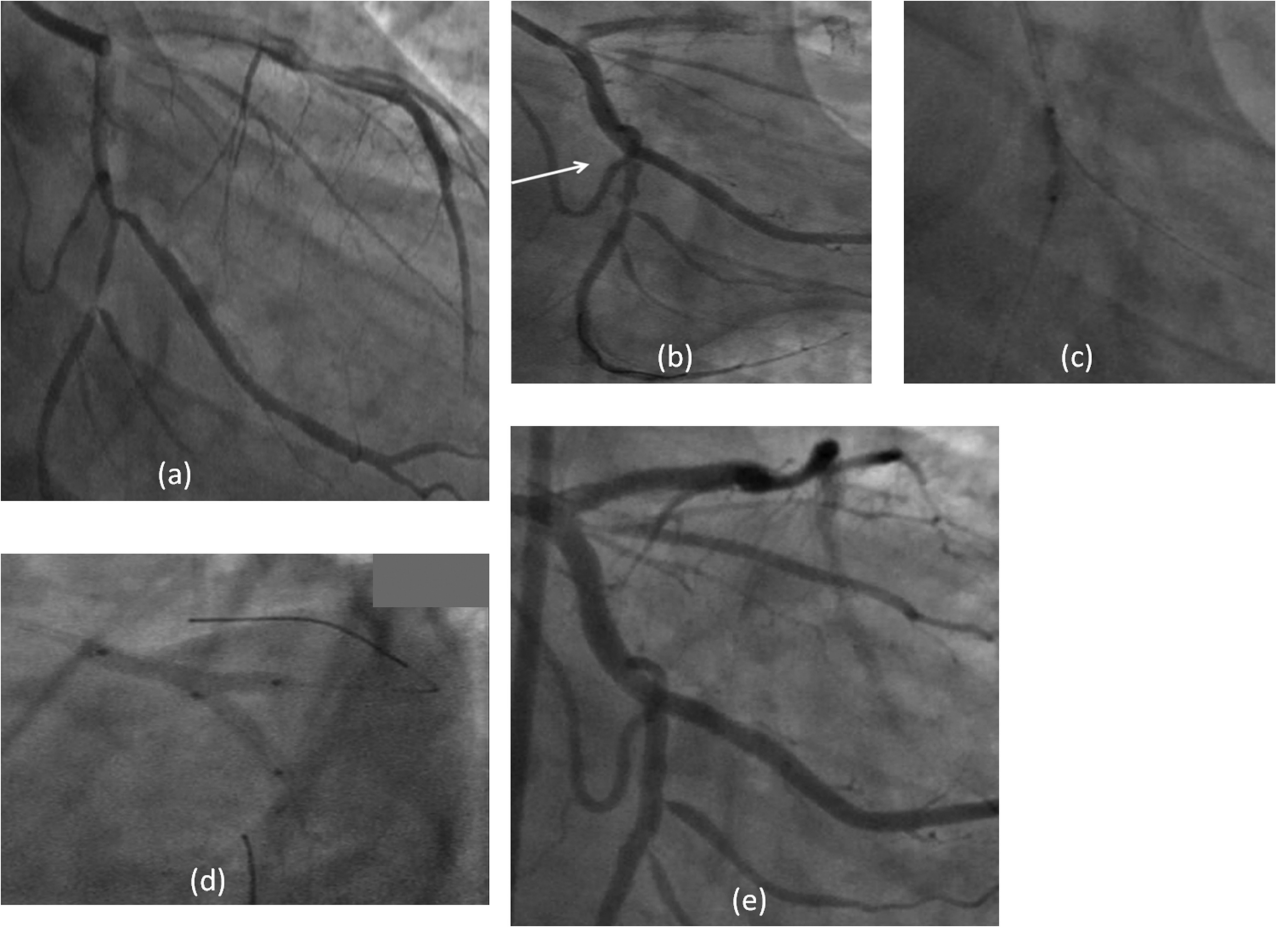
Case 2. (a) Cx, OM1, bifurcation stenosis; (b) 3.0 mm × 28 mm BVS implanted from proximal Cx to OM1. Distal Cx pinched and dissected; (c) Distal Cx dilated through struts of MB BVS; (d) LAO caudal view in Distal Cx and final “snuggle balloon” dilatation after crossing and implantation of a 2.5 mm × 18 mm BVS through the struts of MB BVS; (e) final result.

**Fig. 4:**
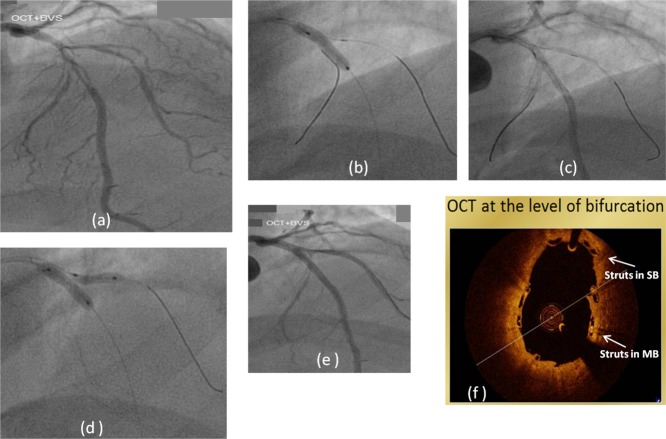
Case 3. (a) AP cranial projection LAD/D1 bifurcation disease; 4(b) 3.0 mm × 18 mm BVS in LAD; (c) TIMI-I flow in D1 after BVS implantation; (d) “snuggle balloon” dilatation following 2.5 mm × 28 mm BVS implanted into D1 through the struts of MB BVS; (e) final result; (f) OCT image at the level of bifurcation.

The technique to salvage SB followed in our other two cases is similar and we have represented it in Figs. 3 and 4 and tabulated the salient features in Table 1.

**TABLE 1:** Summary of angiographic and procedural detail for all patients

Case/Figure no.	Vessels involved in bifurcation disease	Medina classification	Visually estimated diameter and % stenosis of involved vessels	SB protected by wire and dilated	MB BVS size (mm) post dilation (PD)│balloon size│pressure	Status of SB after MB BVS implantation	SB BVS Size (mm) Post Dilation (PD)│balloon size│pressure	Strategy used	Final simultaneous snuggle balloon dilatation	Final OCT of MB & SB	Complications	Clinical follow-up (months)
Case I (Fig. 1)	LAD and D1	1, 1, 1	LAD: 3.0 mm, 80%; D1: 2.5 mm, 80%	Yes	BVS: 3.0 × 28 mm; PD: 3.0 mm at 20 atm	• Pinched 90% at osteum with dissection	2.5 × 18 mm; PD: 2.5 mm at 20 atm	Provisional “TAP”	Yes at 8 atm.	Yes (Fig. 2)	None	10-months asymptomatic
						• Fractional flow reserve (FFR) not measured						
Case 2 (Fig. 3)	Cx and OM1	1, 0, 1	Cx/OM1: 3.0 mm, 70%; Cx/OM2: 2.5 mm, 80%	Yes	BVS: 3.0 × 28 mm; PD: 3.25 mm at 24 atm	• Dissection with 70% residual stenosis	2.5 × 18 mm; PD: 2.5 mm at 20 atm	Provisional “TAP”	Yes at 6 atm.	No	None	8-months asymptomatic
						• Fractional flow reserve (FFR) not measured						
Case 3 (Fig. 4)	LAD and D1	1, 1, 1	LAD: 3.0 mm, 80%; D1: 2.5 mm, 90%	Yes	BVS: 3.0 × 28 mm; PD: 3.0 mm at 22 atm	• Closure with TIMI-I flow	2.5 × 28 mm; PD: 2.5 mm at 20 atm	Provisional “TAP”	Yes at 8 atm.	Yes (Fig. 4)	None	9-months asymptomatic
						• Fractional flow reserve (FFR) not measured						

## DISCUSSION

Bifurcation lesions with an important SB reportedly occur in nearly 20% of the patients undergoing PCI [3]. However, experience of treating bifurcation lesions using BVS is extremely limited and unreported. Lesions involving SB > 2 mm have been excluded from all previous studies of BVS [4]. Bench testing demonstrates that the cells of the MB BVS can be eased open by slow and graded expansion with a 1.5 mm and 2.0 mm balloon to nominal pressures without causing disruption of the struts. A 2.5 mm balloon dilatation of the cells to nominal pressures (12 atm) may result in an occasional fracture of interconnecting links without loss of integrity or radial strength (Data on file, Abbott Vascular, Santa Clara, USA). A simultaneous final balloon dilatation may be needed to optimize the MB scaffold and bifurcation, though, simultaneous “kissing balloon” dilatation is not recommended due to the theoretical risk of over sizing in the proximal segment of MB BVS leading to mechanical disruption. We therefore conceptualized on a “non kissing” technique of simultaneous balloon dilation termed as “snuggle balloon,” which we describe. Following the “TAP” technique and “snuggle balloon” dilatation, the OCT images in our cases showed uniform and adequate expansion of BVS struts in the MB and SB with good apposition of struts at bifurcation minimal protrusion of the SB BVS struts into the MB at the level of carina (distal edge) and optimal coverage of the proximal edge of the SB ostium without visible geographical miss. There was no over dilatation or disruption of MB BVS proximal to the SB.

From our clinical experience of treating 51 bifurcation lesions with BVS, we have evolved the recommended technical steps to achieve optimal results, while treating a bifurcation lesion with a clinically important SB, where single stent strategy with SB protection and provisional SB stenting may be considered.

- Both MB and SB are wired and the MB is stented with BVS. Following MB stenting, if a clinically significant pinching of SB ostium judged by angiography or fractional flow reserve (FFR), dissection or threatened closure of SB occurs then the struts of the MB BVS are recrossed with a wire into the SB and dilated sequentially using low profile 2.0 mm followed by 2.5 mm diameter balloon gradually inflating the balloon at 2 atm, every 5 sec till full expansion is achieved. Final optimization of the bifurcation is performed using a simultaneous balloon dilation in such a manner that the proximal end of the SB balloon protrudes only marginally in the MB and is dilated first, the main branch balloon is then dilated at moderate pressures (8–10 atm) so as to create an inverted Y i.e. λ (Fig. 3d) with practically no proximal overlap or “kissing.” This technique of simultaneous balloon dilation which is not “kissing balloon” avoids over expansion beyond the recommended limits and hence disruption of BVS proximal to the SB and yet optimizes the result at bifurcation. We have termed this final simultaneous dilatation as “snuggle balloon dilatation” (snuggle is defined as “to lie or curl up together closely and comfortably”). Subsequent in vitro bench testing [5] also supports this strategy.
- In case of threatened closure of SB, a 2.5 mm BVS is eased through the struts of MB BVS. Gentle sustained pressure and manipulation of the SB wires may help to get the BVS through. If unsuccessful, the BVS is withdrawn cautiously and replaced with a low profile metallic DES. Once the BVS crosses into the SB, it is deployed keeping the proximal stent delivery balloon marker just proximal to the MB ostium, so that the BVS proximal marker falls at the ostium (Fig. 1d). This ensures a 0.8 mm or less protrusion of the scaffold into the MB almost bordering a T-stenting (micro TAP technique). After optimization of the SB and MB BVS using sequential dilation with high pressure NC balloons keeping within the recommended limits of expansion as per IFU, a final simultaneous “snuggle balloon dilation” is performed at moderate pressures to optimize the result.

The provisional single stent strategy in these cases was chosen based on accepted wisdom from previous data that in metallic DES the results of a single stent strategy are equal if not superior to a two stent strategy and is also less complex. There is also no data, recommendations, or even case reports of the choice of technique or results of two stent strategies using BVS for bifurcation lesions.

While our experience is preliminary, all our three attempts to get the BVS into SB through the struts of MB BVS have led to successful results and no complications on a relatively short-term follow-up. This till now has been unreported. At this stage we only recommend the TAP technique using two BVS as a “bailout” strategy for significant residual stenosis or threatened closure of SB following treatment of MB by BVS. However, the application of this technique as an elective two stent strategy for treating bifurcation lesion with BVS, could be the subject of future studies as more and longer term experience is gained in “real world” patients. We ourselves plan to study these patients by CT angiography at 1-year and by coronary angiography with OCT at 2-years and 3-years.

## CONCLUSION

Through these three illustrative cases, we have demonstrated that the bifurcation lesions with a large SB need not be a contraindication to the use of BVS. SB access, optimization, and salvage using a provisional “TAP technique” to deploy a second BVS through the struts of MB BVS is feasible provided meticulous attention to technical details as described are followed. Simultaneous “snuggle balloon dilation” may be important for result optimization when using BVS for bifurcation disease without causing disruption of MB BVS.
